# Software for web-based tic suppression training

**DOI:** 10.12688/f1000research.13460.2

**Published:** 2018-08-06

**Authors:** Jonathan K. Black, Kevin J. Black

**Affiliations:** 1 Ira A. Fulton College of Engineering and Technology, Brigham Young University, Provo, UT, 84602, USA; 2 Washington University School of Medicine, St. Louis, MO, 63110, USA

**Keywords:** behavior therapy, software, tic disorders, Tourette syndrome, reward

## Abstract

Exposure and response prevention (ERP) is a first-line behavior therapy for obsessive-compulsive disorder and Tourette syndrome (TS). However, ERP for tic disorders requires intentional tic suppression, which for some patients is difficult even for brief periods. Additionally, practical access to behavior therapy is difficult for many patients, especially those in rural areas. The authors present a simple, working web platform (TicTrainer) that implements a strategy called reward-enhanced exposure and response prevention (RE–ERP). This strategy sacrifices most expert therapist components of ERP, focusing only on increasing the duration of time for which the user can suppress tics through automated differential reinforcement of tic-free periods (DRO). RE–ERP requires an external tic monitor, such as a parent, during training sessions. The user sees increasing digital rewards for longer and longer periods of successful tic suppression, similar to a video game score. TicTrainer is designed with security in mind, storing no personally identifiable health information, and has features to facilitate research, including optional masked comparison of tics during DRO *vs.* noncontingent reward conditions. A working instance of TicTrainer is available from https://tictrainer.com/

## Introduction

Recent years have seen increasing evidence for and acceptance of behavior therapies for tic disorders such as Tourette syndrome ( Capriotti *et al.*, 2014). Tic suppression plays a key role in these therapies ( Specht *et al.*, 2014). One of these is exposure and response prevention (ERP), a first-line treatment for obsessive-compulsive disorder ( Verdellen *et al.*, 2011). In a randomized, controlled trial, ERP showed similar tic reduction efficacy to habit reversal therapy, the most extensively studied behavior therapy for tics, with a mean 8.6-point decrease in the Yale Global Tic Severity Scale (YGTSS) after 12 two-hour ERP sessions ( Verdellen *et al.*, 2004). However, tic suppression, an essential component of ERP, is difficult or frustrating for some tic patients. Fortunately, we showed that even children with recent-onset tic disorders could suppress tics when brief tic-free periods were rewarded immediately by small tokens ( Greene *et al.*, 2015). Our experience with that study, and in an unrelated project, suggested to us that automating the process of immediate rewards for tic-free intervals might facilitate ERP, even in those who at first could suppress tics only for a few seconds at a time ( Black *et al.*, 2017; Miller *et al.*, 2015). There is substantial evidence that contingent reward enhances tic suppression without producing a rebound effect, at least for periods of time up to 30–40 minutes ( Brabson *et al.*, 2016; Conelea *et al.*, 2018; Himle *et al.*, 2007; Himle *et al.*, 2008; Specht *et al.*, 2013; Woods *et al.*, 2008). Within a single session, contingently reinforced tic suppression produced decreases in tic frequency whether or not the participants were directed to attend to premonitory urges ( Specht *et al.*, 2013). However, whether repeated practice of tic suppression leads to sustained, clinically relevant improvement is not known, because unlike typical ERP, tic suppression alone does not explicitly encourage attention to the premonitory urge (the “exposure” part of ERP).

Here we present a simple, web-based tool to facilitate training intended to allow increasing periods of tic suppression. Design goals included using this program to record tics, to provide rewards for tic suppression in a video-game-like format that many children would be familiar with, to gradually increase rewards for increasing periods of tic suppression, to respect confidentiality, to gather anonymous information that can be used to assess use patterns and initial information about efficacy and safety, and to provide features that facilitate research use, *i.e.* creation of research subject accounts that can be assigned to different reward schedules at different points in time.

## Methods

### Implementation

The TicTrainer server is written in JavaScript, using the Node.js runtime.

### Account registration and anonymity

The system is designed to ensure security of users’ personally identifying information. Instead of recording a name, each user is assigned a simple account ID used to log on. To track the collective age of users and to allow potential individualized training dependent on age, the user is asked his or her birth month and year. However, the system saves only a randomly chosen birthdate within 45 days of the 15th day of the month supplied.

Administrative accounts also can be created. Admins can flag certain users as research participants and assign them to receive rewards differently from regular users. They can also view system and session log files, or create another admin account.

### Storing data

Each user and trainer account has its own text-based account data file on the server. This is a simple way to store modest amounts of data without using a database, at the possible expense of keeping numerous files open simultaneously if traffic to a single site becomes very heavy. System design may need to change if the number of users increases very substantially.

### Logging on

A user’s ID, password, and other account information are sent back and forth as needed between client and server. When a user types his ID and password into the “Manage Account” sign in page, for example, the server sends back a unique web page that includes the credentials, as he/she provided them, and some information loaded from his/her account data. If he/she edits something in his/her account, those same credentials are sent back to the server for authentication along with the new data.

Any page on the website that has information specific to a signed-in user is created dynamically by the server. These dynamic web pages are stored as .dynh files on the server. (“.dynh” is a custom extension which stands for DYNamic Html.) These files have designated locations for the server to inject the required unique fields (usually these include the user’s ID and password) before sending the edited page back to the client. Almost every page involved in a training session is stored and generated this way by the server, so as to preserve the user’s credentials as he/she typed them at the beginning of the session.

During a session, the server mediates between the user and trainer via a session log file. When the trainer records a user’s tic, the trainer’s page sends an XMLHttpRequest asking the server to write a line to the log file. On the other side, the user’s page continually checks the session log file on the server for changes, so it can reset the rewards when the user tics and end the session when the trainer leaves. At the end of a session, the log file is archived with the end time in the filename.

### Operation

TicTrainer runs on a node.js server. Users need a web browser that supports javascript. The program may not function properly on browsers that do not support HTML5, and currently does not work with Microsoft browsers.

### Setting up

A visitor to TicTrainer first registers an account. User and trainer accounts are created separately. Either one next goes to the “Manage Account” page and links to another account. Specifically, trainers specify the users they train, and users specify the trainers that can train them.

### Training sessions

During a session, the trainer is presented with two buttons: “Tic Detected,” and “End Session.” When “Tic Detected” is pressed, the server logs a tic. Trainers also see a 1-minute timer progressing continuously next to an “I’m Here” button, which restarts that timer (as does the “Tic Detected” button). If the trainer presses “End Session,” closes the page, or lets the “I’m Here” timer elapse, the session ends. The timer helps ensure that the trainer stays engaged in watching and recording the user’s tics.

The user’s session page displays a large counter for their current point total, followed by a superscript number indicating the current point accrual rate. For each session, the reward point rate starts at zero, and the rate resets to zero after each tic. Each time the user refrains from ticcing for a number of seconds equal to his/her level, their point total increases by the current rate, and the rate then increases by the square of the current level (rate is capped at 10 × *levels* ^2^). Finally, a user “levels up” when his/her points exceed 1000 × *levels* ^2^. He/she then also receives “coins” equal to the square of the previous level. The coins can be traded in for digital medals at an online store. Parents or clinicians may choose to provide tangible rewards for the digital coins or medals. The medals are displayed during training sessions and on the user’s “Manage Account” page. In total, this reward strategy is intended to provide users increasing incentives to suppress their tics for increasingly long intervals.

For potential use in controlled trials, research participant accounts can also be assigned to an alternative (control) reward strategy, noncontingent reward ( Greene *et al.*, 2015; Himle *et al.*, 2008). In this case the admin user can set the initial mean frequency of rewards. This frequency may be set based on the participant’s previously recorded tic frequency, to better mask the treatment allocation. Thereafter the frequency of rewards increases automatically based on the subject’s achieved “level,” with the intention of approximately matching reward frequency with the two methods.

## Use cases

**Figure 1.**
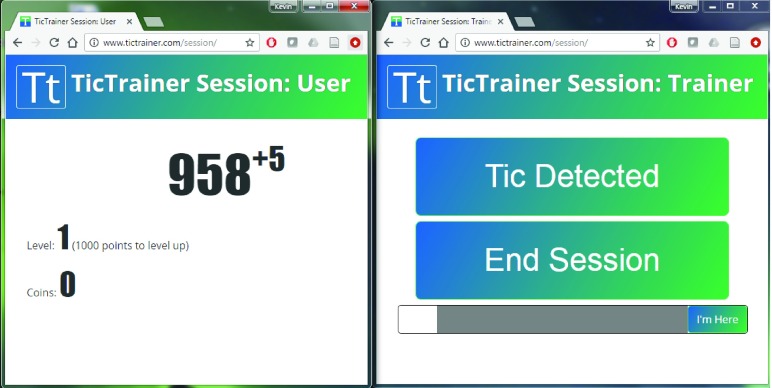
Screenshots from a TicTrainer.com session. Left: the “user” window (person with tics). Right: the “trainer” window (clinician or other trained observer). Typically the two windows would appear on separate devices.

Figure 1 shows a “user” window (for the person with tics) and a “trainer” window as they might appear during a session. In typical use, the two windows would appear on two separate devices (but they can be opened on the same device, as shown here). This user is currently on level one, with 958 points, and is accruing 5 points for every second during which no tics are detected. This user has not yet earned any coins.

Supplementary File 1 is a session log file for a test session (no human subjects were observed). Admin users can view or download these session log files, from which they can compute for each session any of the following:

- measures of tic frequency and tic suppression, *e.g.*
  ◦ mean frequency of tics
  ◦ longest tic-free interval
  ◦ number of 10-second tic-free intervals
- number of rewards
- other metrics, *e.g.*
  ◦ tests of whether inter-tic intervals fit a fractal pattern ( Peterson & Leckman, 1998)
  ◦ tests of the timing of tics vis-à-vis timing of rewards

## Conclusions

This simple web-based platform is available at TicTrainer.com, and provides features that allow prospective trials including with different reward schedules. Features that could yet be implemented include adding self-report (and/or trainer-report) measures of inter-session tic severity or of other symptoms, or measures of premonitory sensations/urge intensity before, during or after sessions ( Brandt *et al.*, 2016; Himle *et al.*, 2007; Specht *et al.*, 2014; Verdellen *et al.*, 2008). Showing the user’s personal record times (maximum achieved tic-free duration) would be another feature of interest.

Limitations of the software include the following. A “trainer” is required to monitor tics, yet neither machines nor humans are perfect tic monitors. The process of monitoring tics itself can reduce tic rate. The data recorded for the timing of tic occurrence does not distinguish motor vs. phonic tics, face vs. extremity tics, or simple vs. complex tics. This choice was in part driven by the senior author’s experience monitoring tics for research, in that tracking individual tics separately in real time is impracticable for most patients. Additionally, while habit reversal therapy and its descendant Comprehensive Behavioral Intervention for Tics focus on one or two tics at a time, traditional ERP, like this software, focuses on tic suppression overall. The software itself does not encourage attention to premonitory urges, a focus of traditional ERP. One can provide instructions to do so outside of the software, or use the software without explicit exposure instructions in order to test whether ERP’s efficacy depends on this component. Finally, this approach has not yet been tested for either tolerability or efficacy in tic patients.

## Software availability

TicTrainer available from: https://tictrainer.com/

Source code available from https://github.com/jonkb/TicTrainer-node

Archived source code as at time of publication: http://doi.org/10.5281/zenodo.1325945 ( Black, 2018).

License: MIT

## Supplementary material

**Supplementary File 1: Sample regular log file.** The log file from a sample training session. This test user account was not flagged for research, so this log file demonstrates the typical reward strategy (differential reinforcement of other behavior, or DRO).
