# Supplementary material for: Software for web-based tic suppression training
Source: F1000Res. 2018 Aug 6;6:2150. Originally published 2017 Dec 19. [Version 2] doi: 10.12688/f1000research.13460.2 (PMC6117856; doi:10.12688/f1000research.13460.2)
Supplement: Peer review history

# Peer review: Referee response for version 2

**DOI:** 10.5256/f1000research.17288.r36851

**Published:** 2018-09-10

**Authors:** Davide Martino

**Affiliations:** 1 Department of Clinical Neurosciences, Cumming School of Medicine, Hotchkiss Brain Institute, University of Calgary, Calgary, AB, Canada

The authors' revisions have substantially improved the article and addressed my initial comments. I don't have further suggestions for improvement.

I have read this submission. I believe that I have an appropriate level of expertise to confirm that it is of an acceptable scientific standard.

# Peer review: Referee response for version 2

**DOI:** 10.5256/f1000research.17288.r36852

**Published:** 2018-09-04

**Authors:** Cara W.J Verdellen

**Affiliations:** 1 PsyQ Nijmegen, Nijmegen, Netherlands

I have read the revisions with interest and noticed that the authors adequately addressed the questions raised.

I have read this submission. I believe that I have an appropriate level of expertise to confirm that it is of an acceptable scientific standard.

# Peer review: Referee response for version 2

**DOI:** 10.5256/f1000research.17288.r36853

**Published:** 2018-08-30

**Authors:** Christine A. Conelea

**Affiliations:** 1 Department of Psychiatry, University of Minnesota, Minneapolis, MN, USA

The authors have been responsive to reviewer feedback and adequately addressed the questions raised.

I have read this submission. I believe that I have an appropriate level of expertise to confirm that it is of an acceptable scientific standard.

# Peer review: Referee response for version 1

**DOI:** 10.5256/f1000research.14616.r30608

**Published:** 2018-03-15

**Authors:** Cara W.J Verdellen

**Affiliations:** 1 PsyQ Nijmegen, Nijmegen, Netherlands

TicTrainer is developed to support exposure and response prevention (ERP) in the behavioral treatment of tics. ERP is considered a firstline intervention for tic disorders according to European Guidelines (Roessner et al; Verdellen et al, ESSTS, 2011). Patients are encouraged to suppress tics for prolonged periods of time allowing them to get used to unpleasant premonitory urges that often accompany tics. TicTrainer is a very valuable, nice and simple tool that supports the patient in practicing ERP at home, also in the absence of a therapist. It is also based on the finding that tic suppression is enhanced by reinforcement of tic-free periods. TicTrainer stores anonymous information to assess use patterns and information about efficacy and safety. This makes TicTrainer a promising software tool for both clinical and research ends.

 Unfortunately, I was unable to evaluate the website itself because after creating an account I did not manage to make it work (‘Input Error: improper ID’). Therefore, my review is based only on the manuscript. I recommend the authors to pay attention to these ‘’technical problems’’, and also to get it supported by Microsoft browsers, as this may be a major limitation for usage. 

 TicTrainer can be used to enhance tic suppression ability (response prevention of tics), but in its present form, it does not include the element of exposure to premonitory urges. Since exposure is an important part of ERP (learning to tolerate the urge, not give in to it by performing tics), it is highly recommended to integrate this element, for example by adding a measure of sensations/urges.

 More literature demonstrating the effect of direct reinforcement on tic suppression should be included in the introduction as it supports the rational for this tool in enhancing tic suppression ability.

 It is described in this manuscript that ‘’preferably’’ two devices are needed; one for the patient and one for the trainer, who can be a parent/partner. It doesn’t say if it also works on one device (do you see 2 windows?). A disadvantage of two devices may be that a patient always needs another person to practice with, which is not always possible. It is suggested to consider integrating both tic detection and suppression/reinforcement in one tool/device (like the app BT-Coach, see bt-tics.com/bt-coach).

 For what age is this tool applicable?

 Is it right that a patient doesn’t see his tic suppression (record) times on the window, only ‘’levels’’ and ‘’earned coins’’? It may be encouraging and reinforcing to also receive feedback on the exact tic suppression (record) times (as described in the protocol of ERP).

 Since the tool is based on receiving rewards for tic suppression, perhaps more details can be given about what kind of rewards can be used; an example of a rewarding system could be useful. Perhaps ‘’mini’’ games can be integrated in the tool that can be done directly after practicing, depending on the number of coins that are earned/levels achieved?

 Overall I think this is a very promising tic suppression tool!

I have read this submission. I believe that I have an appropriate level of expertise to confirm that it is of an acceptable scientific standard, however I have significant reservations, as outlined above.

# Response

**Published:** 2018-08-02

**Authors:** Kevin J Black

**Affiliations:** Department of Psychiatry, Washington University in St Louis, USA

TicTrainer is developed to support exposure and response prevention (ERP) in the behavioral treatment of tics. ERP is considered a firstline intervention for tic disorders according to European Guidelines (Roessner et al; Verdellen et al, ESSTS, 2011). Patients are encouraged to suppress tics for prolonged periods of time allowing them to get used to unpleasant premonitory urges that often accompany tics. TicTrainer is a very valuable, nice and simple tool that supports the patient in practicing ERP at home, also in the absence of a therapist. It is also based on the finding that tic suppression is enhanced by reinforcement of tic-free periods. TicTrainer stores anonymous information to assess use patterns and information about efficacy and safety. This makes TicTrainer a promising software tool for both clinical and research ends.

Unfortunately, I was unable to evaluate the website itself because after creating an account I did not manage to make it work (‘Input Error: improper ID’). Therefore, my review is based only on the manuscript. I recommend the authors to pay attention to these ‘’technical problems’’, and also to get it supported by Microsoft browsers, as this may be a major limitation for usage. 

**The web site is working now. Steps for registering and starting the first session were originally quite complicated. We now provide careful, step-by-step instructions for both “users” and “trainers,” and we believe this also increases the likelihood of successful use.**

TicTrainer can be used to enhance tic suppression ability (response prevention of tics), but in its present form, it does not include the element of exposure to premonitory urges. Since exposure is an important part of ERP (learning to tolerate the urge, not give in to it by performing tics), it is highly recommended to integrate this element, for example by adding a measure of sensations/urges.

**In the edited introduction and conclusion, we now discuss this as a potential limitation, as a possible future improvement, and as an opportunity to test whether explicit exposure (focus on the urge) is essential to efficacy in ERP of tics (or whether by contrast tic suppression practice itself, if scheduled and repeated, can lead to benefit). This last possibility seems the more plausible given that habituation’s relevance to ERP efficacy is now in doubt.**

**The relevant portions of the revised text include: “**There is substantial evidence that contingent reward enhances tic suppression without producing a rebound effect, at least for periods of time up to 30-40 minutes (Himle *et al.*, 2007; Himle *et al.*, 2008; Woods *et al.*, 2008; Specht *et al*., 2013; Brabson *et al.*, 2016; Conelea *et al.*, 2018). Within a single session, contingently reinforced tic suppression produced decreases in tic frequency whether or not the participants were directed to attend to premonitory urges (Specht *et al*., 2013). However, whether repeated practice of tic suppression leads to sustained, clinically relevant improvement is not known, because unlike typical ERP, tic suppression alone does not explicitly encourage attention to the premonitory urge (the “exposure” part of ERP). … The software itself does not encourage attention to premonitory urges, a focus of traditional ERP. One can provide instructions to do so outside of the software, or use the software without explicit exposure instructions in order to test whether ERP’s efficacy depends on this component. **”**

More literature demonstrating the effect of direct reinforcement on tic suppression should be included in the introduction as it supports the rational for this tool in enhancing tic suppression ability.

**Done: “**There is substantial evidence that contingent reward enhances tic suppression without producing a rebound effect, at least for periods of time up to 30-40 minutes (Himle *et al.*, 2007; Himle *et al.*, 2008; Woods *et al.*, 2008; Specht *et al*., 2013; Brabson *et al.*, 2016; Conelea *et al.*, 2018). Within a single session, contingently reinforced tic suppression produced decreases in tic frequency whether or not the participants were directed to attend to premonitory urges (Specht *et al*., 2013). **”**

It is described in this manuscript that ‘’preferably’’ two devices are needed; one for the patient and one for the trainer, who can be a parent/partner. It doesn’t say if it also works on one device (do you see 2 windows?). A disadvantage of two devices may be that a patient always needs another person to practice with, which is not always possible. It is suggested to consider integrating both tic detection and suppression/reinforcement in one tool/device (like the app BT-Coach, see bt-tics.com/bt-coach).

**We now address this question in “Use cases”: “**Figure 1 shows a “user” window (for the person with tics) and a “trainer” window as they might appear during a session. In typical use, the two windows would appear on two separate devices (but they can be opened on the same device, as shown here). **”**

**We feel that BT-Coach is a wonderful (and more mature) application. However, the TicTrainer approach grew out of our recent study on children with recent onset of tics, who often are less likely to notice their own tics. We feel that tic monitoring by a third party may be a feature rather than a drawback in certain use cases.**

For what age is this tool applicable?

**We don’t know for certain but expect that anyone who could sit through a session of even 10 minutes or so may be able to use it.**

Is it right that a patient doesn’t see his tic suppression (record) times on the window, only ‘’levels’’ and ‘’earned coins’’? It may be encouraging and reinforcing to also receive feedback on the exact tic suppression (record) times (as described in the protocol of ERP).

**Correct, though the increasing reward rate with increasing duration of tic suppression (through 10 seconds initially, or 10 ×** ***level* seconds later) is a weak proxy. We agree that the record times would be a potentially useful addition. We have added to conclusion the following: “**Showing the user’s personal record times (maximum achieved tic-free duration) would be another feature of interest. **”**

Since the tool is based on receiving rewards for tic suppression, perhaps more details can be given about what kind of rewards can be used; an example of a rewarding system could be useful. Perhaps ‘’mini’’ games can be integrated in the tool that can be done directly after practicing, depending on the number of coins that are earned/levels achieved?

**That is a great idea that at this time is beyond our skill. One benefit of our releasing this code is that others with time and skill beyond ours can add to it.**

Overall I think this is a very promising tic suppression tool!

**Thank you!**

# Peer review: Referee response for version 1

**DOI:** 10.5256/f1000research.14616.r30850

**Published:** 2018-03-05

**Authors:** Davide Martino

**Affiliations:** 1 Department of Clinical Neurosciences, Cumming School of Medicine, Hotchkiss Brain Institute, University of Calgary, Calgary, AB, Canada

The concept of the TicTrainer is highly valuable. Unfortunately I was unable to review the current version of the TicTrainer website directly due to issues with registration (it won’t accept ID – if crucial for this peer review, please provide some guidance in a Supplementary file or in the reply). In general, the authors should comment whether there is a plan in place to overcome the inability of Microsoft browsers to support the program, as this may be a major limitation for diffusion. 

1. Like the other reviewer, I also think that the Introduction is too simplistic in comparing efficacy between ERP and CBIT, whereas in fact the quality of the evidence supporting these two behavioral treatments is different, and has been systematically reviewed and even meta-analysed. A short paragraph summarizing the evidence supporting the efficacy of ERP, also with respect to CBIT, would be useful.
2. Moreover, the Introduction should help a reader who is not fully acquainted with ERP understanding the technique of this behavioral approach, particularly the way in which trainer and patient interact during treatment sessions, and how this type of interaction is suitable to be operationalized using a software tool. All this is a bit given for well established in the paper, whereas in fact the literature on alternative modes of administering ERP is scant.
3. Perhaps I am missing some basic characteristic of the TicTrainer software, but from this manuscript it is not clear whether the “user” window and the “trainer” must appear on separate devices, or whether they could be opened on the same computer simultaneously. Can the authors comment on whether this is a specific choice of the authors of the software (and why), or a technical limitation that aims to be overcome in software upgrades?
4. Finally, I think that the lack of premonitory urge measures is an important limitation. On an individual patient basis, the lack of efficacy of contingent reward application to ERP might depend on the intensity of the premonitory urges and by an insufficient habituation to the urge. If the urges are not measured somehow, it would be difficult to monitor treatment response. A recent paper by Brandt et al., Cortex 2006 proposes a real time, computer-based urge monitoring technique; it would be interesting to integrate/implement something similar in this software.

I have read this submission. I believe that I have an appropriate level of expertise to confirm that it is of an acceptable scientific standard, however I have significant reservations, as outlined above.

# Response

**Published:** 2018-08-02

**Authors:** Kevin J Black

**Affiliations:** Department of Psychiatry, Washington University in St Louis, USA

The concept of the TicTrainer is highly valuable. Unfortunately I was unable to review the current version of the TicTrainer website directly due to issues with registration (it won’t accept ID – if crucial for this peer review, please provide some guidance in a Supplementary file or in the reply).

**The web site is working now. Steps for registering and starting the first session were originally quite complicated. We now provide careful, step-by-step instructions for both “users” and “trainers,” and we believe this also increases the likelihood of successful use.**

In general, the authors should comment whether there is a plan in place to overcome the inability of Microsoft browsers to support the program, as this may be a major limitation for diffusion. 

**This is a quite reasonable concern, but we have not yet identified the specific problem. However, since other free browsers for MS Windows, Apple OS, Linux, Android and Chrome OS are in very common use, we suspect this will not constitute a real barrier to its use.**

1. Like the other reviewer, I also think that the Introduction is too simplistic in comparing efficacy between ERP and CBIT, whereas in fact the quality of the evidence supporting these two behavioral treatments is different, and has been systematically reviewed and even meta-analysed. A short paragraph summarizing the evidence supporting the efficacy of ERP, also with respect to CBIT, would be useful. 

**I agree that the evidence for efficacy of ERP is less solid than for CBIT, but ERP advocates reasonably point to the Verdellen, 2004, trial comparing it to HRT, and their own experience. In any case, a long discussion seems out of place in a short paper introducing software. We added a citation in the introduction to the ESSTS review of behavior therapies for TS.**

2. Moreover, the Introduction should help a reader who is not fully acquainted with ERP understanding the technique of this behavioral approach, particularly the way in which trainer and patient interact during treatment sessions, and how this type of interaction is suitable to be operationalized using a software tool. All this is a bit given for well established in the paper, whereas in fact the literature on alternative modes of administering ERP is scant.

**The introduction now points out that exposure by focusing on premonitory urges is an important part of traditional ERP and we discuss in introduction and conclusion how this approach differs.**

3. Perhaps I am missing some basic characteristic of the TicTrainer software, but from this manuscript it is not clear whether the “user” window and the “trainer” must appear on separate devices, or whether they could be opened on the same computer simultaneously. Can the authors comment on whether this is a specific choice of the authors of the software (and why), or a technical limitation that aims to be overcome in software upgrades?

**The original idea was for Johnny with tics to be seated at a computer or tablet and Mom to be using her smart phone, but it can be done on a single screen. We now specify this in “Use cases”: “**Figure 1 shows a “user” window (for the person with tics) and a “trainer” window as they might appear during a session. In typical use, the two windows would appear on two separate devices (but they can be opened on the same device, as shown here). **”**

4. Finally, I think that the lack of premonitory urge measures is an important limitation. On an individual patient basis, the lack of efficacy of contingent reward application to ERP might depend on the intensity of the premonitory urges and by an insufficient habituation to the urge. If the urges are not measured somehow, it would be difficult to monitor treatment response. A recent paper by Brandt et al., Cortex 2006 proposes a real time, computer-based urge monitoring technique; it would be interesting to integrate/implement something similar in this software.

**We also are very interested in monitoring premonitory urge severity, though that likely would affect outcome. The conclusions now include the following: “**Features that could yet be implemented include adding self-report (and/or trainer-report) measures of … premonitory sensations/urge intensity before, during or after sessions (Brandt *et al.*, 2016; Himle *et al.*, 2007; Specht *et al.*, 2014; Verdellen *et al.*, 2008). **”**

# Peer review: Referee response for version 1

**DOI:** 10.5256/f1000research.14616.r30166

**Published:** 2018-02-02

**Authors:** Christine A. Conelea

**Affiliations:** 1 Department of Psychiatry, University of Minnesota, Minneapolis, MN, USA

This paper describes TicTrainer, a newly created software program designed to track tic occurrences and deliver reward for successful suppression during live observation. Tic suppression contingent reward has reliably been shown to acutely enhance tic suppression in laboratory settings, but little work has been done to date to translate this finding into the clinical context. TicTrainer is an important step in this direction, and appears to be a promising tool for both research and clinical applications. To note, I was unable to evaluate the website itself due to “time out” errors, so my review is based only on the manuscript. Suggestions for enhancing the manuscript are as follows:

1. Introduction: In the sentence, “ERP also showed similar tic reduction..” clarify if the other treatment being referred to is Habit Reversal Therapy/Comprehensive Behavioral Intervention for Tics. It would be helpful to the reader to summarize the outcomes reported for ERP for tics in terms of response rate and degree of change in tic severity reported in prior research.
  A key feature of ERP is that it aims to train tic suppression in the presence of the premonitory urge so that one can habituate to those aversive sensations. This should be noted in the introduction, particularly since this program would train suppression in a manner that is de-linked from the urge.
2. A brief summary of the literature demonstrating the robust, acute enhancement effect of contingent reward on tic suppression should be included in the introduction. This is a consistently replicated finding, which further supports the rationale for creating a more automated reward delivery program.
3. The introduction suggests an intent to utilize data input into the software for research purposes; however, at present the tictimer.com site does not appear to inform users of this intent. It is unclear to me if this is meant as a research only tool at this time, or if anyone can access the site. Clarify whether other possibility identifying information can be collected (e.g., IP address).
4. It seems that each “level” corresponds to a particular reinforcement schedule. Specific details about these schedules and how they change as one progresses through the program would be helpful. I assuming the program is intended to shape longer tic suppression durations but am not clear if the program aims to do this with an increasing or decreasing reward density. It also sounds like the reward schedule can be manipulated in a research context, so clarification of what the default settings vs. customizable settings would be useful.
5. A section discussing limitations of the current iteration of the software would be useful, such as reliance on live observation by the trainer (as tic frequency can be impacted by observation, and real-time judgements about tic occurrences can be difficult). The program only codes tic occurrence dichotomously, which precludes analyses related to tic type (e.g., motor vs. vocal, simple vs. complex).
6. It would be beneficial to clarify whether this software has been tested in those with tics yet.

I have read this submission. I believe that I have an appropriate level of expertise to confirm that it is of an acceptable scientific standard, however I have significant reservations, as outlined above.

# Response

**Published:** 2018-08-02

**Authors:** Kevin J Black

**Affiliations:** Department of Psychiatry, Washington University in St Louis, USA

This paper describes TicTrainer, a newly created software program designed to track tic occurrences and deliver reward for successful suppression during live observation. Tic suppression contingent reward has reliably been shown to acutely enhance tic suppression in laboratory settings, but little work has been done to date to translate this finding into the clinical context. TicTrainer is an important step in this direction, and appears to be a promising tool for both research and clinical applications. To note, I was unable to evaluate the website itself due to “time out” errors, so my review is based only on the manuscript.

**The web site is working now. Steps for registering and starting the first session were originally quite complicated. We now provide careful, step-by-step instructions for both “users” and “trainers,” and we believe this also increases the likelihood of successful use.**

Suggestions for enhancing the manuscript are as follows:

Introduction: In the sentence, “ERP also showed similar tic reduction..” clarify if the other treatment being referred to is Habit Reversal Therapy/Comprehensive Behavioral Intervention for Tics. It would be helpful to the reader to summarize the outcomes reported for ERP for tics in terms of response rate and degree of change in tic severity reported in prior research.

**Yes, the comparison was HRT/CBIT, which had more robust published evidence for efficacy. Since this is a software paper, the introduction was light on detail about ERP. Still, we now provide somewhat more information, as requested.**

A key feature of ERP is that it aims to train tic suppression in the presence of the premonitory urge so that one can habituate to those aversive sensations. This should be noted in the introduction, particularly since this program would train suppression in a manner that is de-linked from the urge.

**We now discuss this potentially important difference in the next-to-last paragraph of the introduction, and briefly mention it again at the end of the conclusion.**

A brief summary of the literature demonstrating the robust, acute enhancement effect of contingent reward on tic suppression should be included in the introduction. This is a consistently replicated finding, which further supports the rationale for creating a more automated reward delivery program.

**Done (next-to-last paragraph of introduction).**

 

1. The introduction suggests an intent to utilize data input into the software for research purposes; however, at present the tictimer.com site does not appear to inform users of this intent. It is unclear to me if this is meant as a research only tool at this time, or if anyone can access the site. Clarify whether other possibility identifying information can be collected (e.g., IP address).
**Anyone can access the site and use the software as is (or create their own instantiation on their own server to change its details and control its use). The program is not intended to track IP address, but servers can do so.**
2. It seems that each “level” corresponds to a particular reinforcement schedule. Specific details about these schedules and how they change as one progresses through the program would be helpful. I assuming the program is intended to shape longer tic suppression durations but am not clear if the program aims to do this with an increasing or decreasing reward density. It also sounds like the reward schedule can be manipulated in a research context, so clarification of what the default settings vs. customizable settings would be useful.               
**Higher levels give higher reward density but reward frequency is slower. We provide the details in the revised ms. (“**For each session, the reward point rate starts at zero, and the rate resets to zero after each tic. Each time the user refrains from ticcing for a number of seconds equal to his/her level, their point total increases by the current rate, and the rate then increases by the square of the current level (rate is capped at 10 × *levels* ^2^). **”**
**In other words, at level 1, the first second without a tic is rewarded with 0 points, the 2nd second without a tic is rewarded by 1 point, and so on until the eleventh (and beyond) consecutive second without a tic is rewarded with 10 points. Once a tic happens, the rate resets to 0 point per second. At level 2, two seconds must pass before the first reward, and the rate then increases to 4 points. After two additional seconds the rate increases to 8 points, and so on to a maximum of 40 points per 2 seconds.** **As for the question about research use, the current version of the software allows administrators to change a participant from the contingent reward schedule above to a noncontingent reward strategy in which rewards are given regardless of tics at a fixed initial rate that then increases with each subsequent level.**
3. A section discussing limitations of the current iteration of the software would be useful, such as reliance on live observation by the trainer (as tic frequency can be impacted by observation, and real-time judgements about tic occurrences can be difficult). The program only codes tic occurrence dichotomously, which precludes analyses related to tic type (e.g., motor vs. vocal, simple vs. complex).
**Done (last paragraph).**
4. It would be beneficial to clarify whether this software has been tested in those with tics yet.
**We’re just offering the software. We don’t yet know whether it will work or how well it is tolerated (though of course we want to find out). Added to last paragraph.**

# Response

**Published:** 2018-08-06

**Authors:** Kevin J Black

**Affiliations:** Department of Psychiatry, Washington University in St Louis, USA

Oops, that sentence should have read:

"Higher levels give higher reward **magnitude**, but reward frequency is slower."
